# Supplementary material for: Single-Cell RNA Transcriptome Profiling of Liver Cells of Short-Term Alcoholic Liver Injury in Mice
Source: Int J Mol Sci. 2023 Feb 22;24(5):4344. doi: 10.3390/ijms24054344 (PMC10002329; doi:10.3390/ijms24054344)
Supplement: Supplementary file 1 [file ijms-24-04344-s001.zip › ijms-2122280-supplementary.pdf]

SUPPLEMENTARY MATERIALS

Single-Cell RNA Transcriptome Profiling of Liver Cells of Short-Term Alcoholic Liver Injury in Mice

Ligang Cao [1], Di Wu [1,2], Lin Qin [1,2], Daopeng Tan [1,2], Qingjie Fan [1,2], Xiaohuan Jia [1], Mengting Yang [1], Tingting Zhou [1], Chengcheng Feng [1], Yanliu Lu [1,2] and Yuqi He [1,2,*]

[1] Guizhou Engineering Research Center of Industrial Key-Technology for Dendrobium Nobile, Zunyi Medical University, Zunyi 563000, China
[2] Joint International Research Laboratory of Ethnomedicine of Ministry of Education, Zunyi Medical University, Zunyi 563000, China
* Correspondence: yqhe2016@zmu.edu.cn

Figure S1. Alcohol consumption causes damage to multiple organs.

Figure S2. Batch effect and Clustering of liver transcriptome in mice with alcoholic liver injury.

Figure S3. Reactome pathway enrichment analysis of top-rank genes for 12 cell types.

Figure S4. The dot plot shows the relative expression changes of genes.

Figure S5. Functional enrichment analysis of transcription factor target genes.

Table S1. Cell and gene expression statistics

Table S2. Cell number of each subset in liver

Table S3. Gene number of consistently up- or down-regulated with prolonged alcohol infusion in cell subsets

Table S4. Gene information of consistently up- or down-regulated with prolonged alcohol infusion in cell subsets

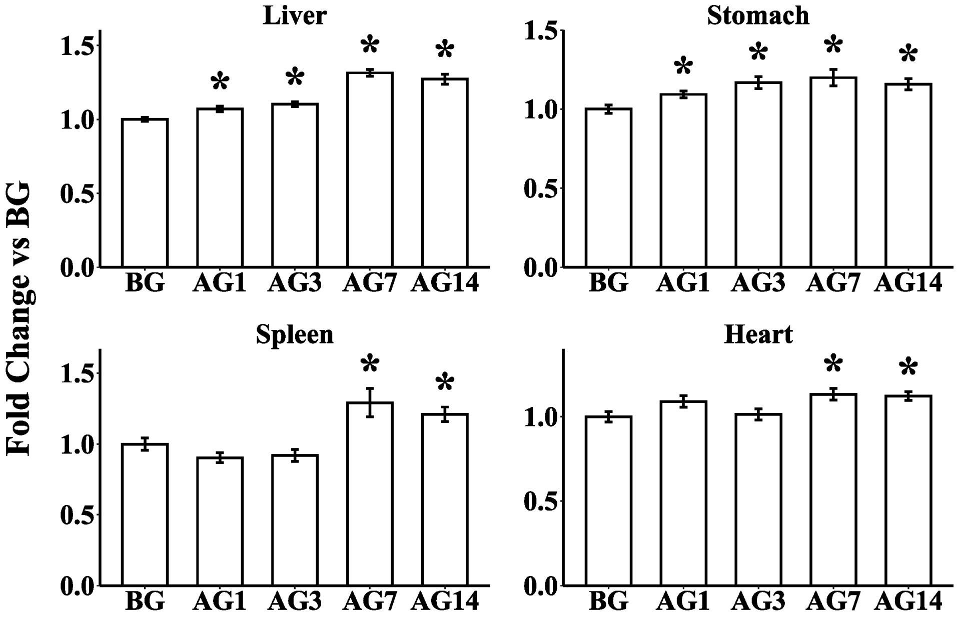

Figure S1. Alcohol consumption causes damage to multiple organs. Liver weight and stomach weight were significantly increased in alcoholic liver injury mice. * P < 0.05 vs BG.

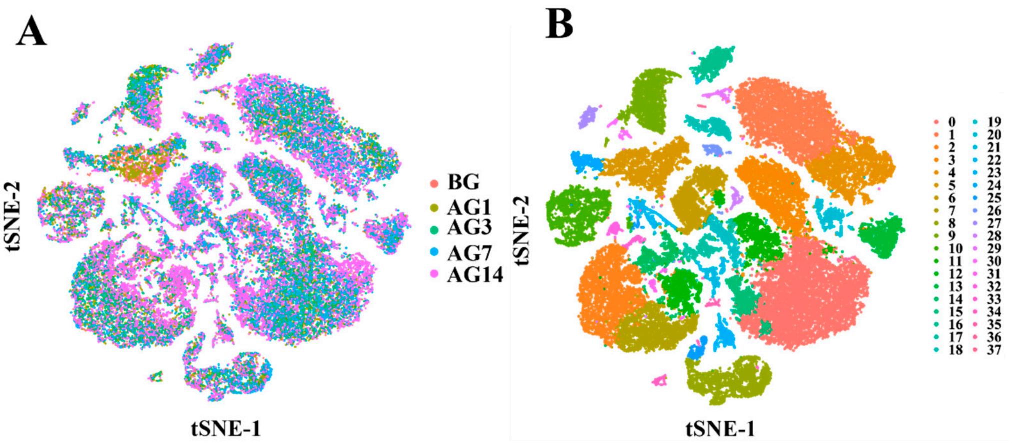

Figure S2. Batch effect and Clustering of liver transcriptome in mice with alcoholic liver injury. (**A**) A total of 51619 mouse liver single cell transcriptome t-SNE in 5 groups of samples. (**B**) The t-SNE visualization showed that the liver cells clustered into 38 cell subpopulations at 0.8 resolution.

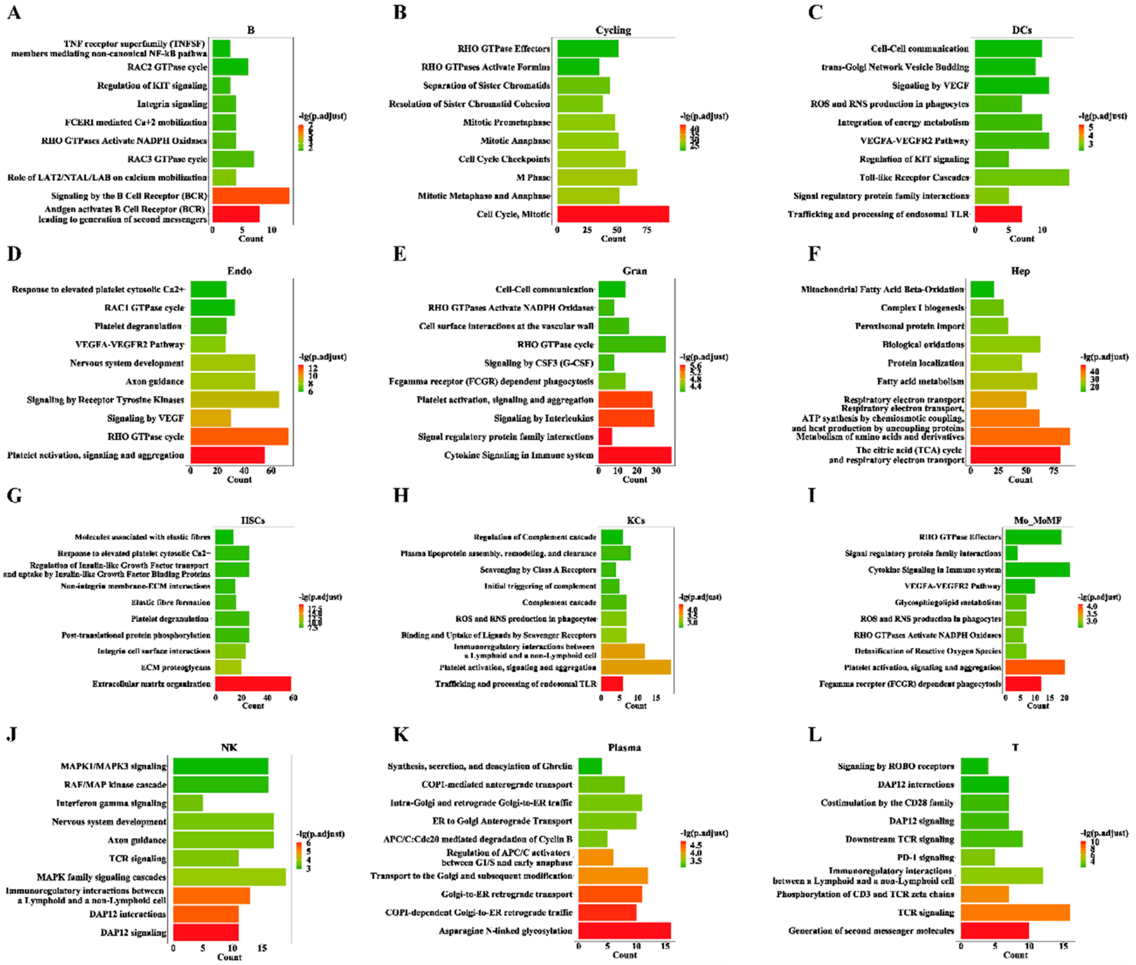

Figure S3. Reactome pathway enrichment analysis of top-rank genes for 12 cell types (B cells, Cycling cells, dendritic cells, endothelial cells, granulocyte, hepatocytes, hepatic

stellate cells, Kupffer cells, monocyte or monocyte-derived macrophages, natural killer cells, plasma cells and T cells).

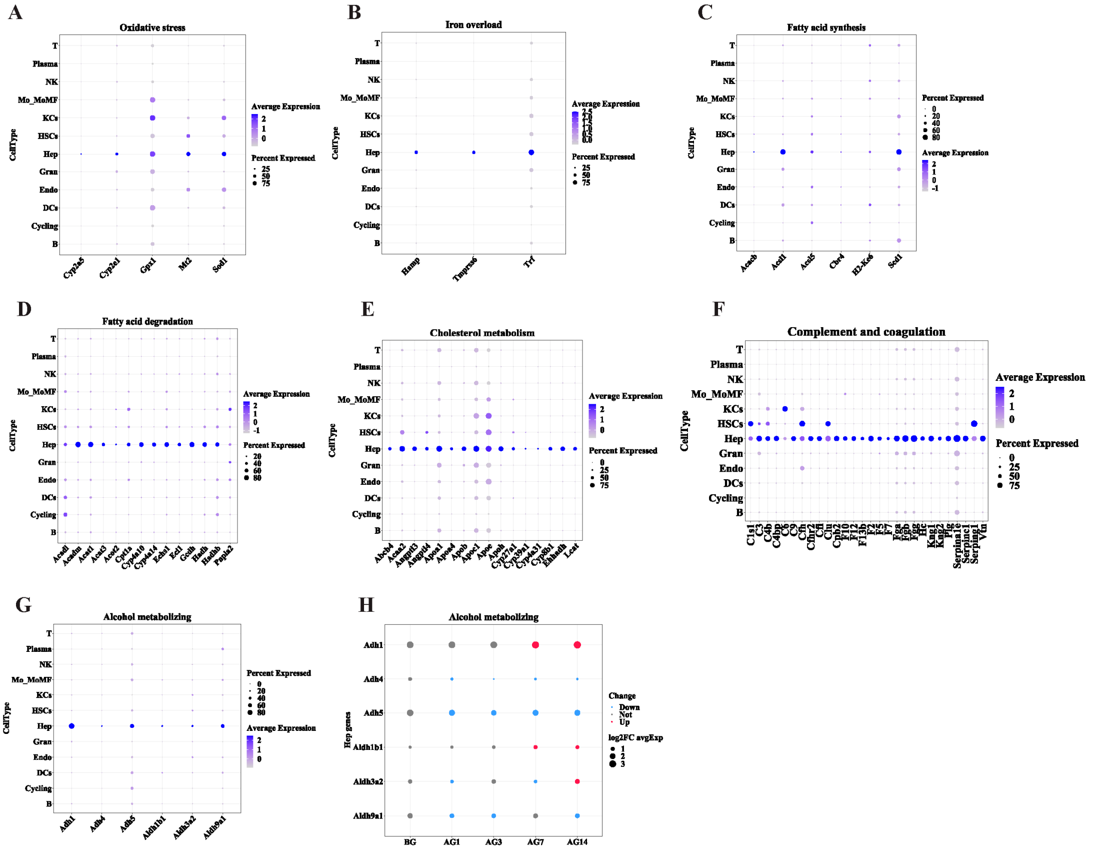

Figure S4. A-G. The dot plot shows the relative expression changes of genes. Expression related to oxidative stress, iron overload, fatty acid synthesis, fatty acid degradation, cholesterol metabolism, complement and blood coagulation, and alcohol metabolizing in different cell types. **H.** Dot map shows the relative expression changes of alcohol metabolizing enzyme genes in different groups of hepatocytes. The size represents the average Log2FC value compared to the BG group.

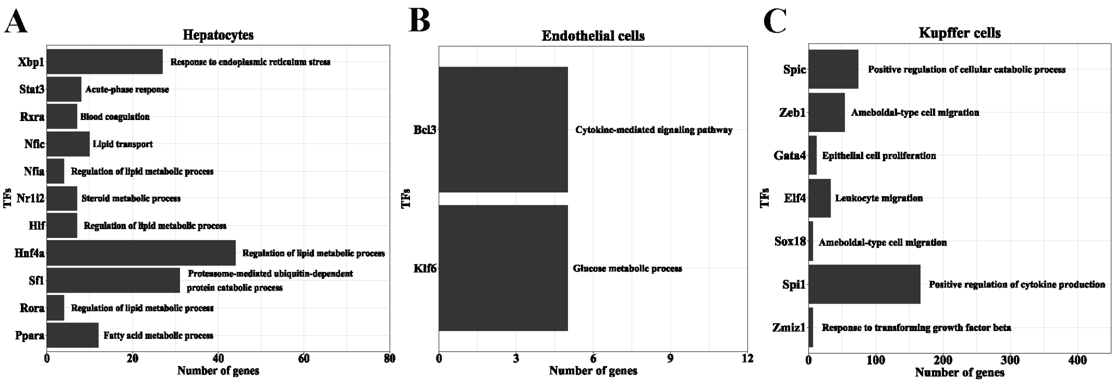

Figure S5. Functional enrichment analysis of transcription factors target genes. (**A**) Bar plots showing the gene ontology (GO) enrichment of TFs target genes in hepatocytes. (**B**) Bar plots showing the gene ontology (GO) enrichment of TFs target genes in endothelial cells. (**C**) Bar plots showing the gene ontology (GO) enrichment of TFs target genes in Kupffer cell. The horizontal axis indicates the number of TF target genes in GO terminology.

Table S1. Cell and gene expression statistics

Sample	Estimated Number of Cells	Fraction Reads in Cells	Mean Reads per Cell	Median Genes per Cell	Total Genes Detected	Median UMI Counts per Cell	Number of cells after filtration
BG	12,427	89.40%	25,629	1,349	19,424	3,231	10439
AG1	13,865	80.30%	30,134	1,350	19,798	3,548	10164
AG3	11,245	87.00%	36,702	1,559	20,136	4,171	9459
AG7	11,504	93.80%	40,780	1,587	19,958	4,632	9260
AG14	12,532	92.60%	36,344	1,677	20,407	4,859	10952

Table S2. Cell number of each subset in liver

Cell number of each subset					
Sample	BG	AG1	AG3	AG7	AG14
B	1900	1446	1566	2059	1900
Cycling	201	54	84	287	145
DCs	200	399	214	195	179
Endo	1463	1648	2117	704	2467
Gran	171	1045	499	303	333
Hep	2369	2048	1177	999	1146
HSCs	42	40	94	17	72
KCs	53	49	130	9	103
Mo/MoMF	252	300	179	180	377
NK	1160	600	692	1050	849
Plasma	151	61	76	189	218
T	2477	2474	2631	3268	3163

Cell number of each subset in liver

Ratio of each subset (%)					
Sample	BG	AG1	AG3	AG7	AG14
B	18.20	14.23	16.56	22.24	17.35
Cycling	1.9255	0.5313	0.8880	3.0994	1.3240
DCs	1.9159	3.9256	2.2624	2.1058	1.6344
Endo	14.0148	16.2141	22.3808	7.6026	22.5256
Gran	1.6381	10.2814	5.2754	3.2721	3.0405
Hep	22.6937	20.1495	12.4432	10.7883	10.4638
HSCs	0.4023	0.3935	0.9938	0.1836	0.6574
KCs	0.5077	0.4821	1.3744	0.0972	0.9405
Mo/MoMF	2.4140	2.9516	1.8924	1.9438	3.4423
NK	11.1122	5.9032	7.3158	11.3391	7.7520
Plasma	1.4465	0.6002	0.8035	2.0410	1.9905
T	23.7283	24.3408	27.8148	35.2916	28.8806

Table S3. Gene number of consistently up- or down-regulated with prolonged alcohol infusion in cell subsets

Gene function	B	Cycling	DCs	Endo	Gran	Hep	KCs	Mo/MoMF	NK	Plasma	T
Acute phase protein	0	0	0	0	0	0	0	0	0	0	0
Alcohol metabolizing	0	0	0	0	0	6	0	0	0	0	0
Apoptosis	0	0	0	7	0	12	5	0	4	2	3
Autophagy	0	0	0	2	0	7	1	0	0	1	0
Cholesterol metabolism	0	0	7	3	0	24	4	0	0	0	0
Complement coagulation	0	0	0	3	0	28	3	0	0	1	0
ERS	0	0	0	1	0	0	0	0	1	1	0
Fatty acid degradation	1	0	0	1	0	14	2	0	1	0	0
Fatty acid synthesis	0	0	0	0	0	6	0	0	0	0	0
Hypoxia	0	0	0	1	0	1	1	0	0	0	0
Iron overload	1	1	1	1	1	3	1	1	1	1	1
MUPs	0	0	0	0	0	5	2	0	0	0	0
NF-kappa B	0	0	1	3	0	1	1	1	2	0	1
Oxidative stress	0	0	0	1	0	5	1	0	0	0	0
TLR	1	1	0	1	0	0	2	1	1	0	1
Total number of genes	3	2	9	24	1	112	23	3	10	6	6

Table S4. Gene information of consistently up- or down-regulated with prolonged alcohol infusion in cell subsets

Gene function	Gene symbol	Cell Type	log_2FC Avg vs BG				
			BG	AG1	AG3	AG7	AG14
Acute phase protein	Crp	Hep	0	0.18	0.53	0.45	0.45
Alcohol metabolizing	Adh1	Hep	0	-0.02	0.02	0.31	0.43
Alcohol metabolizing	Adh4	Hep	0	-0.27	-0.51	-0.39	-0.45
Alcohol metabolizing	Adh5	Hep	0	-0.46	-0.90	-0.51	-0.46
Alcohol metabolizing	Aldh3a2	Hep	0	-0.32	0.10	-0.32	0.38
Alcohol metabolizing	Aldh1b1	Hep	0	0.06	0.15	0.42	0.26
Alcohol metabolizing	Aldh9a1	Hep	0	-0.40	-0.35	-0.24	-0.31
Apoptosis	Ctsh	Hep	0	-0.25	0.21	0.27	0.26
Apoptosis	Ctsl	Hep	0	0.61	1.13	0.97	0.77
Apoptosis	Ctsz	Hep	0	0.14	0.52	0.48	0.33
Apoptosis	Cycs	Hep	0	-0.14	-0.86	-0.94	-0.75

Apoptosis	Ctsb	Hep	0	-0.05	0.47	0.66	0.49
Apoptosis	Ctsd	Hep	0	0.16	0.57	0.57	0.53
Apoptosis	Hras	Hep	0	-0.35	-0.59	-0.46	-0.46
Apoptosis	Map2k2	Hep	0	-0.39	-0.57	-0.39	-0.47
Apoptosis	Psma2	Hep	0	-0.21	-0.66	-0.50	-0.40
Apoptosis	Tuba4a	Hep	0	-0.13	-0.56	-0.49	-0.43
Apoptosis	Hmgb1	Hep	0	-0.52	-0.72	-0.58	-0.59
Apoptosis	Endog	Hep	0	-0.46	-0.92	-0.63	-0.51
Autophagy	Atg3	Hep	0	-0.35	-0.62	-0.61	-0.44
Autophagy	Lamp2	Hep	0	0.26	0.52	0.37	0.36
Autophagy	Gabarapl2	Hep	0	-0.23	-0.41	-0.52	-0.26
Autophagy	Gabarap	Hep	0	-0.19	-0.61	-0.52	-0.40
Autophagy	Map1lc3a	Hep	0	-0.30	-0.58	-0.44	-0.30
Autophagy	Map1lc3b	Hep	0	-0.34	-0.41	-0.33	-0.34
Autophagy	Rab1a	Hep	0	0.41	0.40	0.30	0.35
Cholesterol metabolism	Acaa2	Hep	0	-0.78	-1.03	-0.76	-0.54
Cholesterol metabolism	Apoa1	Hep	0	0.16	0.82	0.98	0.30
Cholesterol metabolism	Apoc1	Hep	0	-0.11	-0.19	-0.33	-0.49
Cholesterol metabolism	Apoh	Hep	0	-0.18	0.59	0.67	0.60
Cholesterol metabolism	Apob	Hep	0	0.34	0.64	0.95	0.62
Cholesterol metabolism	Apoe	Hep	0	-0.57	0.18	0.40	0.42
Cholesterol metabolism	Tspo	Hep	0	-0.58	-0.46	-0.38	-0.48
Cholesterol metabolism	Ehhadh	Hep	0	-1.02	1.03	0.23	0.98
Cholesterol metabolism	Lipa	Hep	0	0.20	0.38	0.38	0.53
Cholesterol metabolism	Vdac2	Hep	0	-0.36	-0.61	-0.62	-0.41
Cholesterol metabolism	Vdac3	Hep	0	-0.30	-0.49	-0.47	-0.36
Cholesterol metabolism	Idi1	Hep	0	0.08	-0.49	-0.25	-0.26
Cholesterol metabolism	Cnbp	Hep	0	-0.32	-0.48	-0.48	-0.33
Cholesterol metabolism	Angptl3	Hep	0	-0.78	-0.60	-0.53	-0.66
Cholesterol metabolism	Vapa	Hep	0	-0.09	-0.33	-0.35	-0.25
Cholesterol metabolism	Cyp8b1	Hep	0	-0.24	0.17	0.34	0.47
Cholesterol metabolism	Lcat	Hep	0	-0.16	0.29	0.52	0.27
Cholesterol metabolism	Angptl4	Hep	0	-0.12	0.31	0.30	0.34
Cholesterol metabolism	Cyp27a1	Hep	0	0.14	0.37	0.11	0.32
Cholesterol metabolism	Apoa4	Hep	0	0.77	1.63	1.68	1.62
Cholesterol metabolism	Abcb4	Hep	0	-0.17	0.22	0.24	0.34
Cholesterol metabolism	Cyp39a1	Hep	0	0.05	0.25	0.28	0.34
Cholesterol metabolism	Cyp4a31	Hep	0	-0.07	0.27	0.48	0.34
Cholesterol metabolism	Slco1a4	Hep	0	0.04	0.16	0.17	0.35
Complement coagulation	F2	Hep	0	-0.03	0.51	0.50	0.61
Complement coagulation	Cpb2	Hep	0	0.43	0.67	0.63	0.27
Complement coagulation	Fga	Hep	0	1.76	1.77	1.18	0.74
Complement coagulation	Fgb	Hep	0	1.25	1.49	0.95	0.48
Complement coagulation	Fgg	Hep	0	1.21	1.37	0.74	0.28

Complement coagulation	Kng1	Hep	0	0.19	0.94	1.04	0.69
Complement coagulation	Plg	Hep	0	0.02	0.58	0.92	0.59
Complement coagulation	Serpinc1	Hep	0	-0.21	0.36	0.69	0.63
Complement coagulation	Serpina1e	Hep	0	-0.32	0.12	-0.50	-0.70
Complement coagulation	C3	Hep	0	0.59	1.44	1.45	0.95
Complement coagulation	Hc	Hep	0	0.40	0.84	1.12	0.56
Complement coagulation	C9	Hep	0	-0.06	0.15	0.49	0.35
Complement coagulation	C4b	Hep	0	0.58	0.75	1.14	0.54
Complement coagulation	Cfh	Hep	0	0.86	1.13	1.41	0.78
Complement coagulation	Cfi	Hep	0	0.20	0.77	0.77	0.51
Complement coagulation	C4bp	Hep	0	0.97	1.22	1.10	0.84
Complement coagulation	Cfhr2	Hep	0	0.10	0.43	0.65	0.54
Complement coagulation	Clu	Hep	0	0.19	0.78	1.05	0.61
Complement coagulation	Vtn	Hep	0	0.12	0.57	1.10	0.57
Complement coagulation	F5	Hep	0	0.09	0.35	0.36	0.30
Complement coagulation	F7	Hep	0	0.19	0.38	0.61	0.50
Complement coagulation	F10	Hep	0	-0.11	0.19	0.63	0.43
Complement coagulation	F12	Hep	0	-0.02	0.39	0.59	0.52
Complement coagulation	F13b	Hep	0	0.06	0.21	0.33	0.32
Complement coagulation	Kng2	Hep	0	0.26	0.65	0.71	0.41
Complement coagulation	C6	Hep	0	0.17	0.24	0.47	0.28
Complement coagulation	C1s1	Hep	0	0.28	0.46	0.69	0.39
Complement coagulation	Serping1	Hep	0	0.08	0.72	0.82	0.46
Fatty acid degradation	Acat1	Hep	0	-0.67	-1.12	-0.93	-0.71
Fatty acid degradation	Acadm	Hep	0	-0.59	-0.47	-0.44	-0.31
Fatty acid degradation	Gcdh	Hep	0	-0.50	-0.81	-0.67	-0.47
Fatty acid degradation	Eci1	Hep	0	-1.01	-1.28	-1.01	-1.12
Fatty acid degradation	Cyp4a10	Hep	0	-0.32	1.20	0.49	0.96
Fatty acid degradation	Cyp4a14	Hep	0	-1.36	0.84	0.21	0.70
Fatty acid degradation	Echs1	Hep	0	-0.53	-0.79	-0.48	-0.47
Fatty acid degradation	Pnpla2	Hep	0	-0.07	0.36	0.33	0.37
Fatty acid degradation	Cpt1a	Hep	0	-0.15	0.72	0.65	0.53
Fatty acid degradation	Acat3	Hep	0	-0.54	-0.78	-0.27	-0.30
Fatty acid degradation	Hadhb	Hep	0	-0.43	-0.25	-0.50	-0.39
Fatty acid degradation	Hadh	Hep	0	-0.63	-0.68	-0.42	-0.28
Fatty acid degradation	Acadl	Hep	0	-0.73	-0.68	-0.63	-0.38
Fatty acid degradation	Acot2	Hep	0	-0.08	0.60	0.05	0.36
Fatty acid synthesis	Acsl1	Hep	0	-0.23	0.74	0.28	0.54
Fatty acid synthesis	Scd1	Hep	0	0.28	0.23	-0.06	1.27
Fatty acid synthesis	H2-Ke6	Hep	0	-0.39	-0.66	-0.47	-0.40
Fatty acid synthesis	Acacb	Hep	0	0.02	0.16	0.15	0.26
Fatty acid synthesis	Cbr4	Hep	0	-0.21	-0.31	-0.22	-0.25
Fatty acid synthesis	Acsl5	Hep	0	0.15	0.52	0.55	0.56
Hypoxia	Cebpa	Hep	0	-0.57	-0.54	-0.32	-0.42

Iron overload	Hamp	Hep	0	-1.80	-1.89	-0.99	-2.47
Iron overload	Trf	Hep	0	-0.31	0.61	0.74	0.34
Iron overload	Tmprss6	Hep	0	-0.19	0.25	0.51	0.47
MUPs	Mup1	Hep	0	0.17	-0.02	-0.40	-0.55
MUPs	Mup7	Hep	0	0.10	-0.23	-0.70	-1.08
MUPs	Mup12	Hep	0	-0.18	-0.31	-0.39	-0.63
MUPs	Mup16	Hep	0	0.13	-0.11	-0.61	-0.70
MUPs	Mup21	Hep	0	0.26	0.05	-0.51	-0.89
NF-kappa B	Csnk2b	Hep	0	-0.39	-0.38	-0.36	-0.27
Oxidative stress	Mt2	Hep	0	1.33	0.06	1.48	0.86
Oxidative stress	Cyp2e1	Hep	0	0.20	0.29	-0.31	0.58
Oxidative stress	Gpx1	Hep	0	-1.07	-1.01	-0.62	-0.58
Oxidative stress	Sod1	Hep	0	0.04	-0.11	0.28	0.46
Oxidative stress	Cyp2a5	Hep	0	0.36	1.40	0.70	1.04
Apoptosis	Actb	Endo	0	-0.46	-0.42	-0.17	-0.46
Apoptosis	Actg1	Endo	0	-0.27	-0.39	-0.34	-0.54
Apoptosis	Tuba1b	Endo	0	-0.76	-0.43	-0.42	-0.56
Apoptosis	Sptan1	Endo	0	-0.36	-0.10	0.08	0.27
Apoptosis	Tuba1a	Endo	0	-0.45	-0.21	-0.56	-0.60
Apoptosis	Bcl2	Endo	0	0.01	0.06	0.42	0.55
Apoptosis	Itpr2	Endo	0	0.04	0.08	0.35	0.32
Autophagy	Ddit4	Endo	0	0.38	-0.09	0.50	0.49
Autophagy	Igf1r	Endo	0	-0.43	-0.64	-0.39	-0.32
Cholesterol metabolism	Apoe	Endo	0	-0.64	-0.38	-0.52	-0.47
Cholesterol metabolism	Cd36	Endo	0	-0.06	0.41	0.16	0.62
Cholesterol metabolism	Lipa	Endo	0	0.08	0.13	0.35	0.34
Cholesterol metabolism	Masp1	Endo	0	-0.42	0.01	0.31	0.32
Cholesterol metabolism	Itgb2	Endo	0	0.04	0.02	0.42	0.32
Cholesterol metabolism	Cd59a	Endo	0	0.24	0.28	0.31	0.32
ERS	Pdia3	Endo	0	0.09	0.16	0.12	0.25
Fatty acid degradation	Pnpla2	Endo	0	-0.19	-0.16	0.42	0.33
Hypoxia	Epas1	Endo	0	-0.18	0.05	0.33	0.46
Iron overload	Hamp	Endo	0	-0.63	-1.02	-1.05	-1.11
NF-kappa B	Prkcb	Endo	0	0.17	0.23	0.23	0.26
NF-kappa B	Lck	Endo	0	-0.05	0.02	0.33	0.25
NF-kappa B	Ltb	Endo	0	0.12	0.10	0.41	0.32
Oxidative stress	Nfe2l2	Endo	0	0.02	0.09	0.23	0.26
TLR	Cxcl9	Endo	0	-0.71	-0.82	-0.54	-0.58
Apoptosis	Ctsl	KCs	0	-0.16	0.61	0.11	1.22
Apoptosis	Fos	KCs	0	0.38	0.87	0.75	1.27
Apoptosis	Jun	KCs	0	-0.08	0.54	0.34	1.07
Apoptosis	Psma2	KCs	0	-0.41	-0.61	-1.21	-0.94
Apoptosis	Tradd	KCs	0	-0.57	-0.50	-0.52	-0.46
Autophagy	Ppp2ca	KCs	0	-0.40	-0.47	-1.20	-0.41

Cholesterol metabolism	Hmgcs2	KCs	0	0.22	-0.41	-1.60	-0.99
Cholesterol metabolism	Cd36	KCs	0	0.04	0.99	1.40	1.26
Cholesterol metabolism	Mylip	KCs	0	-0.57	0.57	0.10	1.17
Cholesterol metabolism	Soat1	KCs	0	-0.09	-0.57	-1.60	-0.60
Complement coagulation	C8g	KCs	0	-0.66	-0.80	-0.85	-0.88
Complement coagulation	Thbd	KCs	0	0.16	1.07	0.35	1.10
Complement coagulation	Cd59a	KCs	0	-0.09	0.56	0.20	1.06
Fatty acid degradation	Acaa1b	KCs	0	-0.60	-0.50	-0.98	-0.64
Fatty acid degradation	Eci1	KCs	0	-0.61	-0.67	-0.74	-0.55
Hypoxia	Epas1	KCs	0	-0.84	0.31	0.66	1.30
Iron overload	Hamp	KCs	0	-1.69	-1.78	-1.88	-1.80
MUPs	Mup7	KCs	0	-0.39	-0.70	-1.27	-0.99
MUPs	Mup11	KCs	0	0.25	-0.70	-2.41	-0.66
NF-kappa B	Csnk2b	KCs	0	-0.13	-0.41	-1.08	-0.39
Oxidative stress	Cat	KCs	0	0.26	-0.35	-1.27	-0.51
TLR	Cxcl10	KCs	0	-0.38	-0.71	-0.62	-0.27
TLR	Ifnar2	KCs	0	-0.78	-0.97	-0.56	-0.89
Apoptosis	Gadd45g	NK	0	0.03	-0.07	-0.21	-0.26
Apoptosis	Ctsb	NK	0	-0.12	0.07	-0.18	-0.26
Apoptosis	Map2k2	NK	0	-0.15	-0.09	-0.13	-0.28
Apoptosis	Nfkbia	NK	0	-0.02	-0.42	-0.14	-0.47
ERS	Dnajc3	NK	0	-0.12	-0.22	-0.22	-0.36
Fatty acid degradation	Eci1	NK	0	-0.05	-0.21	-0.23	-0.35
Iron overload	Hamp	NK	0	-0.47	-1.05	-1.03	-1.21
NF-kappa B	Tnfaip3	NK	0	-0.08	-0.67	-0.19	-0.50
NF-kappa B	Ccl4	NK	0	0.02	-0.65	-0.23	-0.84
TLR	Ccl3	NK	0	0.20	-0.41	0.34	-0.40
Cholesterol metabolism	Hmgcs1	DCs	0	-0.09	-0.17	0.32	0.80
Cholesterol metabolism	Cyp51	DCs	0	-0.09	-0.19	0.05	0.45
Cholesterol metabolism	Fdps	DCs	0	-0.35	-0.32	0.31	0.59
Cholesterol metabolism	Idi1	DCs	0	-0.27	-0.42	0.02	0.39
Cholesterol metabolism	Msmo1	DCs	0	-0.36	-0.40	0.14	0.54
Cholesterol metabolism	Sqle	DCs	0	-0.14	-0.16	0.23	0.49
Cholesterol metabolism	Ldlr	DCs	0	-0.19	-0.19	0.03	0.33
Iron overload	Hamp	DCs	0	-0.49	-0.81	-0.81	-0.97
NF-kappa B	Ccl4	DCs	0	0.36	-0.99	0.78	-1.19
Apoptosis	Hras	Plasma	0	-0.17	-0.09	-0.18	-0.28
Apoptosis	Nfkbia	Plasma	0	-0.23	-0.69	-0.36	-0.47
Autophagy	Map1lc3a	Plasma	0	-0.32	-0.27	-0.31	-0.30
Complement coagulation	Serpina1e	Plasma	0	0.04	-0.34	-0.99	-0.99
ERS	Dnajc3	Plasma	0	-0.22	-0.29	-0.20	-0.27
Iron overload	Hamp	Plasma	0	-0.14	-0.39	-0.36	-0.41
Apoptosis	Actb	T	0	-0.60	-0.28	-0.27	-0.47
Apoptosis	Actg1	T	0	-0.79	-0.41	-0.41	-0.44

Apoptosis	Nfkbia	T	0	-0.29	-0.60	-0.26	-0.55
Iron overload	Hamp	T	0	-0.42	-0.98	-1.05	-1.13
NF-kappa B	Tnfaip3	T	0	-0.22	-0.68	-0.31	-0.53
TLR	Ccl5	T	0	-0.74	-0.91	-0.79	-0.53
Fatty acid degradation	Acaa1b	B	0	-0.33	-0.11	-0.96	-0.35
Iron overload	Hamp	B	0	-0.38	-1.05	-1.16	-1.28
TLR	Ccl5	B	0	-0.78	-0.83	-0.53	-0.38
Iron overload	Hamp	Mo_MoMF	0	-0.41	-0.80	-0.79	-0.76
NF-kappa B	Cxcl2	Mo_MoMF	0	-0.03	-1.06	-0.70	-1.10
TLR	Ccl5	Mo_MoMF	0	-1.93	-0.29	-0.93	-0.96
Iron overload	Hamp	Cycling	0	-0.37	-0.66	-0.69	-0.66
TLR	Ccl5	Cycling	0	0.04	-0.73	-0.62	-0.47
Iron overload	Hamp	Gran	0	-1.25	-1.78	-2.16	-2.00
